# A treatment protocol for giant ventral hernias based on predicted postoperative respiratory function

**DOI:** 10.3389/fsurg.2026.1814887

**Published:** 2026-10-12

**Authors:** F. J. Pérez Lara, F. J. Moya Donoso, R. Zubizarreta Jiménez, E. Ojeda Leiva, F. J. Muñoz Castillo, C. Rueda Cruces, C. Ríos Navas

**Affiliations:** 1 Department of General and Digestive Surgery, Hospital de Antequera, Málaga, Spain; 2 DOXA Microfluidics, Málaga, Spain; 3 Department of Anesthesiology, Hospital de Antequera, Málaga, Spain; 4 Department of Radiology, Hospital de Antequera, Málaga, Spain

**Keywords:** abdominal wall reconstruction, botulinum toxin, CT volumetry, giant ventral hernia, loss of domain, progressive pneumoperitoneum, respiratory function

## Abstract

**Background:**

Giant ventral hernias and loss-of-domain abdominal wall defects remain challenging because reintegration of hernia contents may increase intra-abdominal pressure and impair postoperative respiratory function.

**Methods/protocol:**

We describe a structured institutional protocol that integrates abdominopelvic CT volumetry, the hernia sac/abdominal cavity volume ratio, respiratory function testing during progressive simulated abdominal occupation, preoperative botulinum toxin, progressive pneumoperitoneum, transversus abdominis release and, only in selected high-risk cases, volume transposition.

**Clinical implications:**

The protocol is intended to support individualized surgical planning by estimating both abdominal and respiratory consequences before definitive reconstruction.

**Conclusions:**

This manuscript should be interpreted as a methodological protocol and feasibility proposal rather than a validated predictive clinical model. Prospective validation with standardized endpoints is ongoing and will be required before the thresholds can be considered externally validated.

## Introduction

Ventral hernias are the most frequent complication of abdominal surgery, with a global incidence ranging from 2% to 20% (1).

Large hernias that cannot be reduced to the abdominal cavity may impair the patient's quality of life in many ways, including back pain, respiratory difficulties or cosmetic problems (2). They also pose significant difficulties for the surgeon, complicating closure of the abdominal wall and increasing the risks of increased abdominal pressure, compartment syndrome (3) and respiratory problems (4).

For the purposes of this protocol, the terms are used as follows: a giant ventral hernia is defined according to the European Hernia Society as an abdominal wall defect greater than 10 cm in width; loss of domain refers to a volumetric disproportion in which a relevant proportion of the viscera is chronically located outside the abdominopelvic cavity; and in this manuscript the volumetric risk criterion is expressed as the hernia sac volume/abdominal cavity volume ratio (VHC/VAC), with values above 25% considered potentially relevant for postoperative abdominal hypertension according to the Tanaka concept.

The treatment for giant ventral hernia is still very challenging for general surgeons today. Nevertheless, thanks to better knowledge of the functional anatomy of the abdominal wall and of the pathophysiology of intra-abdominal pressure, various techniques have been developed to repair this type of hernia without compromising abdominal function. For example, preoperative procedures may include the combination of pneumoperitoneum with botulinum toxin [as proposed by groups led by Bueno Lledó in Spain (5, 6) and by Elstner in Australia (7)], and surgical techniques such as the separation of the anterior and posterior components have proven useful.

In our opinion, however, a complete evaluation of respiratory function is an essential component of the protocol for this type of hernia, since action on this type of hernia not only requires control at the abdominal level to avoid abdominal hyperpressure and compartment syndrome, but also calls for strict control of the patient's respiratory status, so that the lung function, after the hernia has been reduced to the abdominal cavity, can be estimated. Such a calculation would provide a basis on which to choose the optimum surgical technique to apply, without compromising abdominal or respiratory function. In view of these considerations, we have designed the protocol described below.

## Material and methods (study protocol)

The protocol we propose (Figure 1) for treating a large ventral hernia is based on three fundamental parameters: abdominal volume, hernia volume, and the post-reduction impact on the patient's respiratory function.

**Figure 1:**
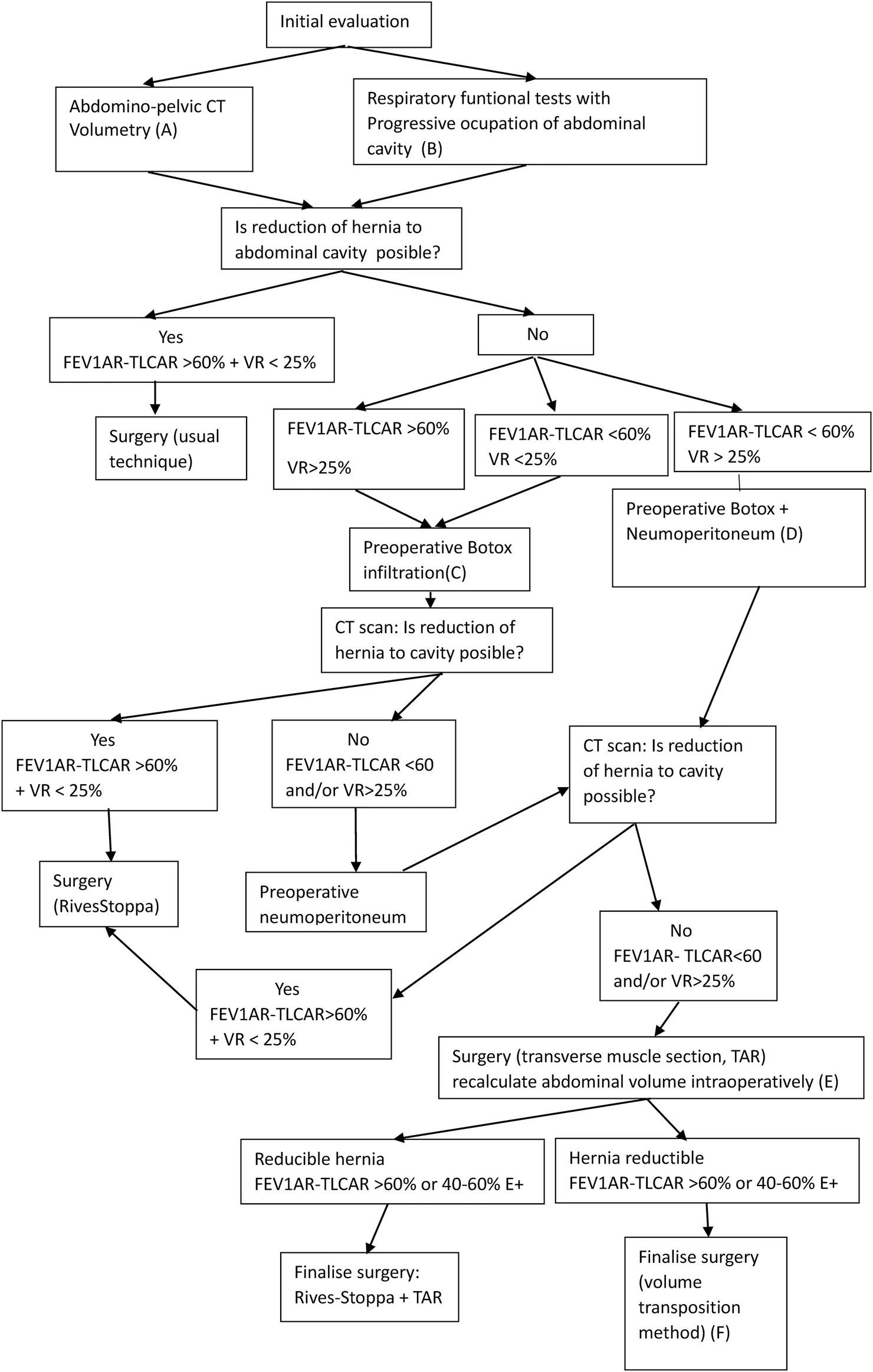
Protocol we propose for treating a large ventral hernia. Abbreviations: FEV1AR (FEV1 after reduction), the FEV1 value simulated by functional tests considering occupation of the abdominal cavity, when the volume of the hernia is reduced; TLCAR (Total Lung Capacity after reduction), the TLC value simulated by functional tests considering occupation of the abdominal cavity, when the volume of the hernia is reduced; VHC/VAC, the relationship between the volume of the hernia and that of the abdominal cavity; E+ (Exercise +), the patient has a FEV1AR value of 40-60% and is able to climb five flights of stairs or walk 400 m without problems; E- (Exercise -), the patient has a FEV1AR value of 40-60% and is not able to climb five flights of stairs or walk 400 m without problems.

### Planned validation

The proposed model will be prospectively evaluated in a consecutive cohort of patients with giant ventral hernias. The planned endpoints include feasibility of complete or partial reintegration, postoperative respiratory complications, need for postoperative ventilatory support, intra-abdominal pressure after closure, abdominal compartment syndrome, surgical-site occurrence, recurrence, bulging, reoperation, mortality, length of stay and follow-up outcomes. Correlation between simulated FEV1AR/TLCAR and actual postoperative respiratory function will be specifically assessed.

### Study design and objective

This article is presented as a study protocol and methodological description. Its objective is to describe a reproducible decision-making pathway for patients with giant ventral hernias or loss-of-domain defects, based on predicted postoperative abdominal and respiratory tolerance after hernia-content reintegration. It is not presented as a definitive clinical validation study.

An initial evaluation should be made of the patient, focusing on respiratory history, previous surgical interventions and current status, together with a detailed physical examination of the abdominal hernia. In addition, an abdomino-pelvic CT scan should be performed, to calculate the volume of the hernia and of the abdomino-pelvic cavity (A), combined with respiratory function tests with progressive occupation of the abdominal cavity (B). These two parameters provide the basis for a preoperative assessment of the abdominal and respiratory repercussions following reduction of the hernia to the abdominal cavity.

Preoperative optimization is considered mandatory before applying the algorithm. This includes smoking cessation whenever possible, weight reduction in patients with obesity, nutritional assessment and correction of hypoalbuminaemia or sarcopenia, optimization of diabetes control, assessment and treatment of COPD and obstructive sleep apnoea, prehabilitation, anaesthetic assessment and individualized thromboembolic prophylaxis. Patients with prohibitive risk despite optimization may be considered for staged reconstruction or non-operative management.

### (A) CT volumetry assessment

The abdomino-pelvic cavity and hernia volumes are calculated as a precautionary measure against compartment syndrome and intra-abdominal hypertension following reduction of the hernia to the cavity.

The volume of the abdomino-pelvic cavity is calculated in accordance with the Tanaka protocol (8), considering the preoperative CT scan of the abdomen, and with the following limits:

The anterior limit, the line connecting the anterior muscle groups of the healthy wall.
The posterior limit, formed by the transverse processes of the vertebrae.
The upper limit, composed of the first axial CT slice, showing the diaphragm.
The lower limit, composed of the final axial CT slice, showing the coccyx.
The lateral parietal peritoneum limits, on each side of the abdominal cavity.

The distances between these limits provide the transverse (d1), anteroposterior (d2) and craniocaudal (d3) diameters, the radii of which are *a* = d1/2, *b* = d2/2 and *c* = d3/2.

The volume of the abdominal cavity can then be calculated using the formula for the volume of an ellipsoid:

v=4/3π×a×b×c

which can be simplified to

4.19×a×b×c

The hernia volume is calculated assuming it takes the form of a hemiellipsoid (9), (because hernia sac protrudes from abdominal wall → modeled as hemi-ellipsoid) where *a* and *b* represent the radii of the length and width, respectively, of the hernia defect of the abdominal wall and *c* represents the height of the hernia. The hernia volume, therefore, is given by the following formula:

(4/3π×a×b×c)/2

Finally, the volume of the hernial cavity is divided by the volume of the abdominal cavity (VHC/VAC). If the result is less than 25%, it is assumed that surgery is feasible and appropriate [Tanaka's group (8) reported no postoperative complications due to occupation, under these conditions].

The ellipsoid and hemiellipsoid formulas are practical bedside approximations and may be less accurate in highly irregular hernia sacs. Whenever available, semi-automated CT segmentation or dedicated volumetric software may provide more precise measurements. Therefore, the mathematical model should be interpreted as an accessible estimation tool rather than a substitute for segmentation-based volumetric analysis.

If the result of the above calculation is greater than 25%, the patient should be prepared preoperatively, with techniques such as Botox infiltration of the abdominal wall (C) and/or progressive pneumoperitoneum (D) in order to prevent intra-abdominal complications.

### (B) Calculating respiratory function with progressive occupation of the abdominal cavity

Functional tests are performed to calculate the patient's preoperative baseline status and the expected impact on respiratory function following reduction of the hernia to the cavity (10). Therefore, all patients with a large abdominal hernia should be given a respiratory function test, performed at baseline. A rigid girdle is then attached, extending from the xiphoid to the pubis (Figure 2) and a 500 cc serum bag is inserted between the girdle and the abdominal wall, thereby restricting the abdominal cavity by this amount. The respiratory function is then measured again. This action is subsequently repeated using serum bags with volumes of 1,000, 1,500, 2,000, 2,500 and 3,000 cc, thus indicating the loss of respiratory capacity that would occur with a given loss of abdominal volume.

**Figure 2:**
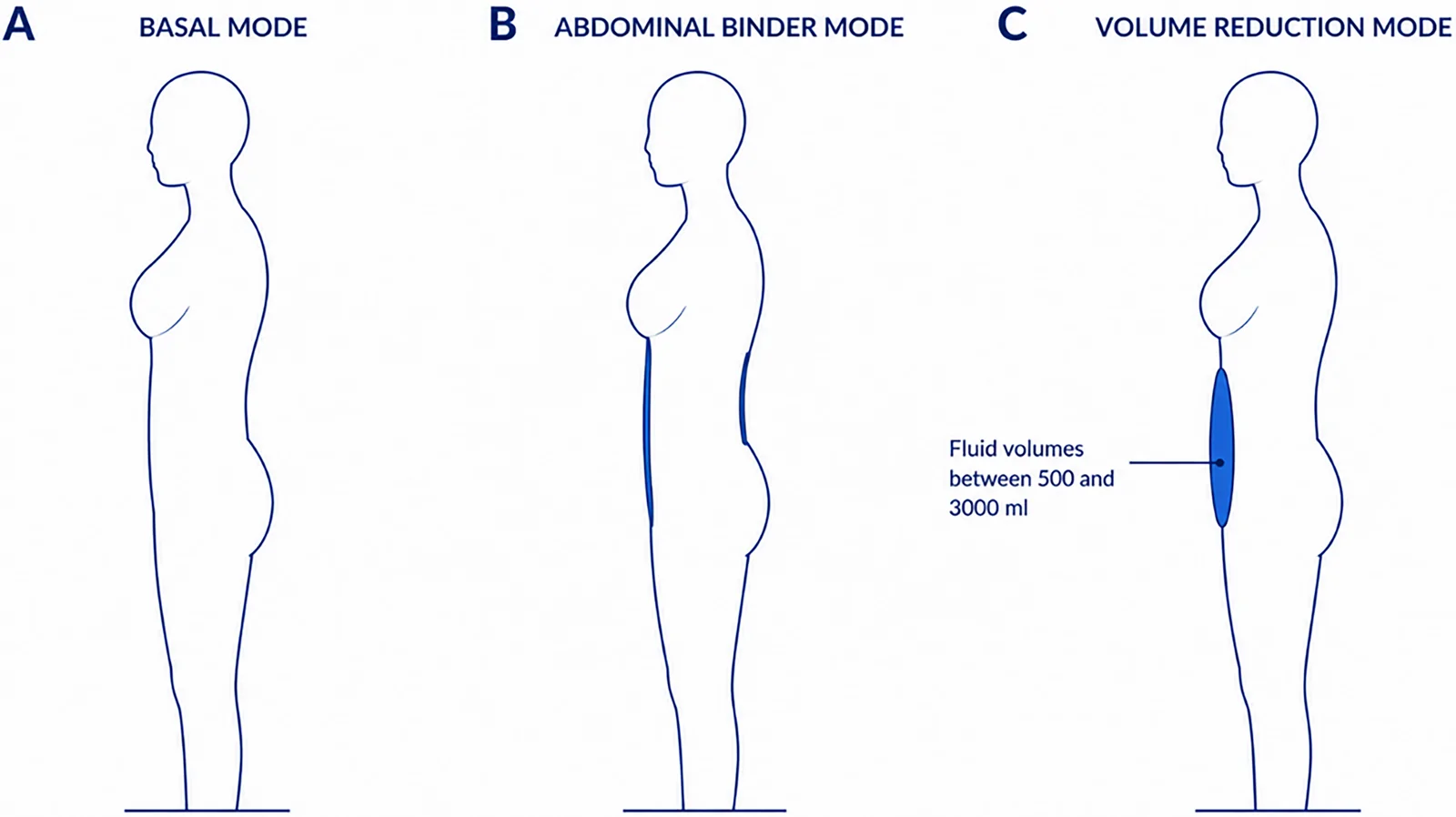
Calculation of respiratory function during progressive simulated occupation of the abdominal cavity. **(A)** Basal mode. **(B)** Abdominal binder mode. **(C)** Volume-reduction mode using fluid bags between 500 and 3,000 mL.

Methodological limitation: the test is innovative but not yet externally validated. The relationship between simulated FEV1AR/TLCAR and actual postoperative respiratory function will be analysed prospectively. Until those data are available, the test should be considered a structured risk-estimation method rather than a validated predictive model.

Standardization of the respiratory simulation test: spirometry is performed with the patient in the same position throughout the assessment, preferably standing or seated according to the pulmonary function laboratory standard used at baseline. The abdominal binder extends from the xiphoid to the pubis and is adjusted to achieve firm but tolerable compression without pain. After each volume increment, a short adaptation period is allowed before repeating spirometry. The same trained operator should perform the test whenever possible, and at least two acceptable manoeuvres are obtained at each step. Patient discomfort, pain, dyspnoea and intolerance are recorded. Obesity, pain, poor cooperation and very large or non-reducible hernias may reduce reproducibility and should be documented.

FEV1 and TLC are the most useful parameters considered in a preoperative assessment. For example, with a hernia that has a volume of 1,500 cc, with this simulation, when the abdominal cavity is limited with a 1,500 cc IV bag, the surgeon will know the FEV1 and TLC values that will be produced after the hernia is reintroduced [henceforth, these values are termed FEV1AR and TLCAR (After Reduction)].

To measure the risk of the surgical intervention, according to the FEV1AR or TLCAR value obtained, we have adapted the risk protocols used by thoracic surgeons in pneumonectomy interventions according to respiratory function (11–15) Thus, the intervention risk is considered low when FEV1AR or TLCAR is >60%, moderate at 40%–60% and high at <40%. This assessment enables us to calculate the maximum volume of hernial content that can be reduced to the abdominal cavity while maintaining FEV1AR and TLCAR >60%. In other words, with hernias that do not exceed this volume the surgeon is assured that the intervention can be performed without prejudice to the patient's respiratory function.

These respiratory thresholds are adapted from thoracic surgical risk stratification and are used here as a conservative reference framework. We acknowledge that abdominal wall reconstruction differs physiologically from lung resection, and that these cut-off values require specific validation in giant ventral hernia patients. For this reason, the thresholds should guide multidisciplinary decision-making but should not be interpreted as independently validated predictors of postoperative respiratory failure in this population.

For a larger hernia volume (FEV1AR or TLCAR <60%), we calculate how much of this volume can be safely reduced (i.e., whilst maintaining FEV1AR and TLCAR >60%) and how much volume remains to be reduced. Thus, a preoperative procedure (such as Botox or pneumoperitoneum) can be applied to expand the abdominal cavity and reduce this remaining volume, whilst maintaining FEV1AR and TLCAR >60% and therefore minimising the risk to the patient.

For cases in which the respiratory functional tests with progressive occupation of the abdominal cavity cannot be performed, we calculate the average respiratory loss presented in our patients for each of the volumes considered derived from institutional cohort and previous publication (10) (Figure 3). Then, from the baseline FEV1 and TLC values, we can calculate the average decrease obtained for the different volumes of abdominal restriction, and so the FEV1AR and TLCAR values can be determined according to the hernia volume to be reduced.

**Figure 3:**
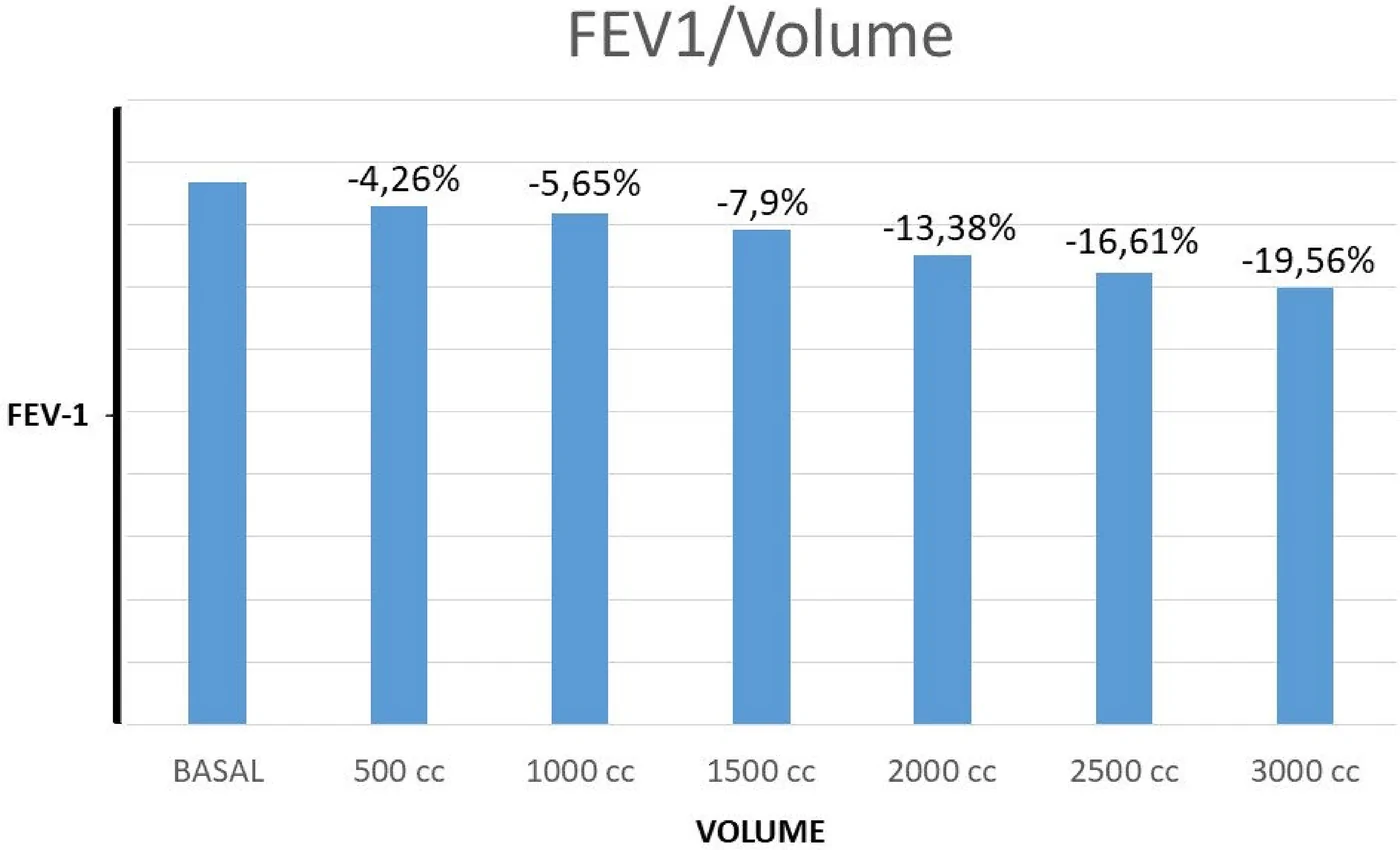
Average respiratory loss presented in our patients for each of the volumes considered.

Having obtained the results for the abdominal cavity and hernia volumes and the FEV1AR-TLCAR values, it is then possible to decide whether it is feasible to reduce the volume of the abdominal hernia, with minimal risk of intra-abdominal hyperpressure (VHC/VAC <25%) and of respiratory repercussions (FEV1AR and TLCAR >60%). If these conditions are met, the usual surgical technique can safely be applied.

The decision pathway is intentionally presented as a detailed algorithmic table rather than as a simplified flowchart. A conventional flowchart would make the sequence easier to read, but it would also omit clinically relevant combinations of volumetric risk, respiratory risk, response to preoperative preparation, exercise tolerance and intraoperative recalculation. For this reason, we have retained a detailed decision algorithm and clarified the thresholds in the accompanying text.

If the above calculations show that the hernia cannot safely be reduced (either because VHC/VAC >25% or FEV1AR-TLCAR <60%), the patient should be prepared with preoperative Botox wall infiltration (C). If both of these unfavourable outcomes are present, the Botox could be supplemented with progressive preoperative pneumoperitoneum (D).

### (C) Abdominal wall infiltration with Botox

The application of botulinum toxin to the lateral muscles of the abdominal wall can significantly increase the average length of these muscles, by about 2.8 cm bilaterally.

The benefit of this neurotoxin arises from the resulting decrease in lateral tension forces on the hernial defect and the elongation of the lateral abdominal muscles, with a subsequent increase in the volume of the abdominal cavity, thus facilitating tension-free abdominal reconstruction.

Muscle paralysis is achieved during the first 10 days and the maximum effect is reached after 3–4 weeks, with a decreasing persistence during the following 6–9 months. The neurotoxin is therefore applied on an outpatient basis at 4–6 weeks before hernia repair surgery.

The points at which the Botox injection should be made are located by ultrasound, visualising the three lateral muscle layers (external oblique, internal oblique and transverse muscles) to be infiltrated. For this purpose, the patient is placed in a lateral position, as recommended by Ibarra-Hurtado (16). Two infiltration points are located on the mid-axillary line, equidistant from the costal margin and the iliac crest, and another three are located on the anterior axillary and mid-clavicular line between the costal margin and the superior iliac crest. This method is repeated on the contralateral side of the abdomen. An injection of 5 mL of Dysport® (50 units/5 mL) or 3 mL of Botox® (30 units/5 mL) is administered at each point.

### (D) Establishing preoperative pneumoperitoneum

In 1947, Goñi Moreno (17) described a technique to achieve the progressive distension of the abdominal cavity, relating lung volume to abdominal volume for a tension-free closure. The method consists of progressively introducing air into the abdominal cavity, thereby increasing its volume, but without increasing its pressure, thanks to the progressive nature of the process.

In the protocol we propose, the treatment sessions consist of the daily insertion of 500–1000 cc for 7–14 days. The total amount is adjusted in accordance with the hernia volume; however, there is no clear consensus in the literature regarding the exact volume to be insufflated or the proportion of gas that remains prior to surgery. Published series describe variable protocols based on patient tolerance and progressive adaptation of the abdominal cavity (4, 5).

In our protocol, the estimation that approximately 15% of the insufflated volume remains if surgery is performed within seven days after the last insufflation is derived from our institutional experience, based on radiological reassessment and intraoperative findings. This value should therefore be interpreted as an approximate clinical reference rather than a fixed physiological parameter, as gas reabsorption rates may vary depending on individual patient factors and timing of surgery.

For example, in a patient with an estimated hernia volume of 1,200 cc, a cumulative insufflation of approximately 8,000 cc may be required to achieve adequate abdominal expansion, provided that this is well tolerated. To enable the insufflation, the puncture is performed under local anaesthesia, using a central line catheter fitted with a particle filter and introduced into the peritoneal cavity via the left hypochondrium.

During this entire insufflation period, the patient receives a prophylactic dose of heparin.

Following the Botox and/or pneumoperitoneum preparation, a further control CT scan is performed to assess how much abdominal volume has thus been gained. If this assessment suggests that the hernia can safely be reduced (VHC/VAC <25% and FEV1AR-TLCAR >60%), the Rives-Stoppa technique (18) is then applied. Otherwise if VHC/VAC >25% and/or FEV1AR-TLCAR <60%, during surgery it would be necessary to apply the lateral expansion technique, with transversus abdominis release (TAR) and then calculating, intraoperatively, the volume gained by this disinsertion. To facilitate this calculation, we have produced a table showing how much the volume of an ellipsoid increases as its anterior wall is advanced (E). If this table shows that, after performing the TAR, the hernial volume reduction would generate a FEV1AR-TLCAR value >60%, then the intervention can safely be completed. Otherwise, and as a last resort, a volume transposition intervention (F) should be considered. With this intervention, mesh remains in contact with the abdominal contents. Therefore, we also consider a moderate-risk subgroup, for whom the Rives-Stoppa intervention (18–20) is appropriate. The patients in this subgroup would have FEV1AR-TLCAR values of 40%–60% and be able to climb five flights of stairs or walk 400 m without problems [Exercise+(E+)]. The volume transposition technique would therefore be indicated only in medium-risk patients (FEV1AR-TLCAR <60% Exercise - (E-) and in high-risk patients (FEV1AR-TLCAR < 40%).

### (E) Intraoperative calculation of volume gain with transversus abdominis release

As stated above, in cases in which after applying Botox and creating pneumoperitoneum, the preoperative CT scan shows that the necessary volume has not been achieved, (FEV1AR or TLCAR is <60%), then a bilateral transverse abdominal release [TAR (21)] must be performed to expand the abdominal volume. It is important to determine the volume gained by this manoeuvre, to know whether the total hernia can be accommodated whilst maintaining good or moderate respiratory function. The corresponding values are shown in Figure 4.

**Figure 4:**
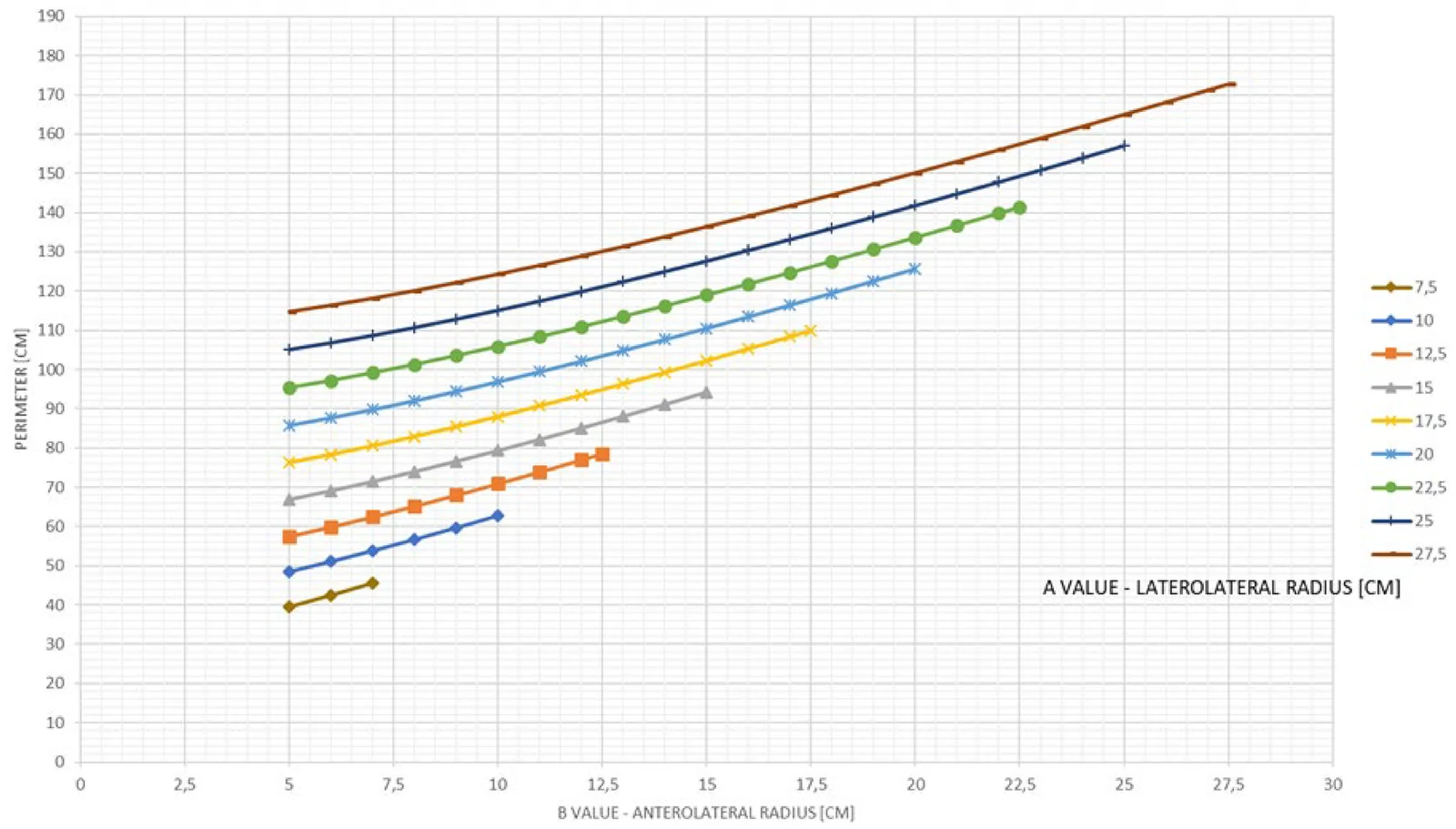
Table to determine intraoperatively the volume gained by TAR manoeuvre.

This table is needed because it is not possible, mathematically, to calculate the increase in volume resulting from an increase in the perimeter of an ellipsoid. This problem is well known, and in 1914 the mathematician Ramanujan (22) proposed an approximation for this purpose, which has a very small margin of error (<1%).

If volume of abdominal cavity is:

v=4/3π×a×b×c

where *a* is the laterolateral radius, *b* is the anteroposterior radius and *c* is the craniocaudal radius of the ellipsoid of the abdominal cavity.

In Figure 4, the value “a” determines the colour of the line chosen. We then examine where the value “b” intersects with this colour line. This intersection indicates the perimeter of the axial cut of the cavity (on the vertical axis of ordinates). To this perimeter value, we then add the number of centimetres gained with the bilateral TAR (measured in the operating room, as the sum of the bilateral distances between the transverse and the rectum after disinsertion). The perimeter value thus obtained is cross-referenced in the table with the chosen colour line, the result of which is the new anteroposterior radius (“b2”) (on the horizontal abscissa axis). We can then recalculate the volume of the cavity generated by TAR, using the new value “b2” (“a” and “c” remain unchanged).

Therefore, the final formula for calculating the volume of the cavity obtained after TAR is:

v=4/3π×a×b2×c

### (F) Volume transposition technique

This option (9) is not intended to replace anatomical abdominal wall reconstruction. It is reserved as a rescue strategy for highly selected patients in whom complete reintegration would generate unacceptable respiratory or abdominal risk despite preoperative preparation and TAR. The indication must be individualized and discussed within a multidisciplinary team.

This technique is used when, despite increasing the size of the abdominal cavity with Botox and/or preoperative pneumoperitoneum, and after performing TAR during the intervention, we calculate that the volume of the hernia would put the patient at important risk.

In this context, the technique is particularly indicated in patients with loss-of-domain hernias and high surgical risk, in whom complete anatomical reconstruction is not feasible or would result in unacceptable respiratory compromise.

In selected patients without signs of incarceration, this approach must be weighed against a non-operative strategy. However, watchful waiting does not prevent progressive enlargement of the hernia, worsening abdominal wall dysfunction, or deterioration in quality of life.

In such cases, the abdominal cavity must be expanded, by replacing part of the anterior abdominal wall with a mesh.

This approach represents a bridging reconstruction, in which the hernia sac is not fully reduced and is instead contained within a prosthetic neowall.

For these reasons, the volume transposition technique should be considered only when the alternative is either unsafe complete reduction or non-operative management with progressive loss of function. Long-term follow-up is required, and the recurrence risk should be explained to the patient during informed consent.

Mesh selection and position must prioritize visceral protection. In our protocol, a prosthesis suitable for intraperitoneal contact or a composite/barrier mesh is used when the mesh may face abdominal contents. The overlap, fixation and double-crown suturing are planned according to the calculated residual volume and the dimensions of the defect. The expected disadvantages of this strategy include a higher risk of bulging, recurrence, adhesions, mesh-related complications and long-term failure compared with anatomical retromuscular reconstruction.

Although bridging techniques have been associated with higher recurrence rates compared to anatomical repair, in this specific subgroup the primary goal is to avoid respiratory failure and excessive intra-abdominal pressure, prioritizing functional safety over anatomical restoration.

The dimensions of the mesh are determined according to the cavity expansion required to maintain good respiratory function (FEV1AR-TLCAR >60%).

### Operative technique

First, we must determine the hernial volume that cannot be reintroduced without provoking unacceptable risk to respiratory function. For example, consider a hernia that has a volume of 939 cc and assume that the FEV1AR-TLCAR value is <40% after pneumoperitoneum, Botox and TAR, and that only 500 cc can be reinserted whilst maintaining FEV1AR-TLCAR >60%. Therefore, 439 cc must be transposed to the new space.

This space is created by the mesh and has the shape of a cylinder with an ellipsoid base. Its volume is calculated as follows:

v=π×a×b×c

where “a” is the longest radius, “b” is the shortest and “c” is the height (Figure 5).

**Figure 5:**
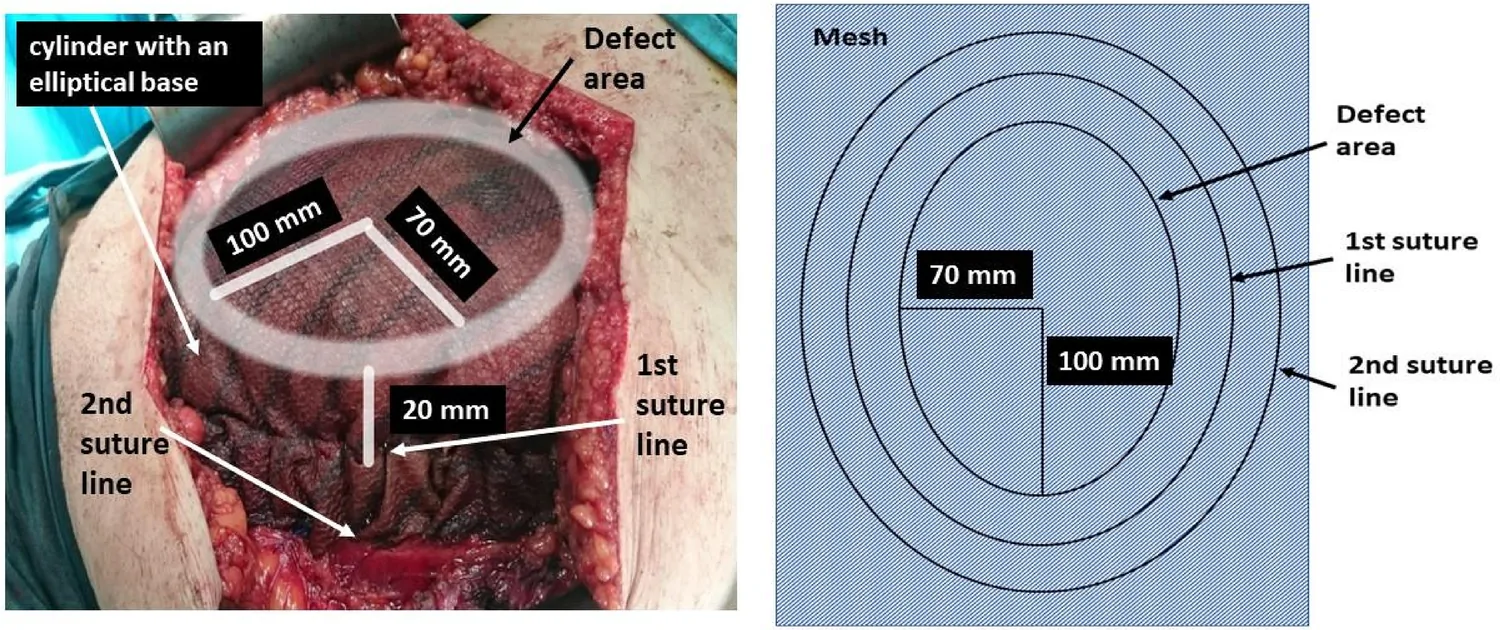
Volume transposition technique. The mesh is prepared with three concentric ellipses. The area enclosed by the innermost ellipse constitutes the roof of the repair. The area between the first and second ellipses determines the height of the reconstructed segment, whereas the area between the second and third ellipses is used for placement of the double-crown fixation sutures.

Accordingly, the mesh is cut into an elliptical shape, with three concentric ellipses sufficient to contain the 439 cc excess volume. For example, suppose the abdominal wall defect measures 10 cm × 7 cm. Then π × 10 × 7 × c = 439 cc, and by determining “c” we establish that the mesh must have a height of 2 cm. Thus, we create a cylindrical structure with an ellipsoid base that acts as an abdominal neowall, containing the 439 cc of hernial volume and preventing postoperative respiratory compromise.

The first suture line joins the edge of the defect to the second ellipse of the mesh. The second suture line provides reinforcement, at 5 cm from the edge, and joins the aponeurosis to the third ellipse of the mesh (double crown technique). The distance between the first and second ellipses constitutes the height of the created space.

### (G) Measuring intra-abdominal pressure

To confirm the accuracy of our calculations, an intravesical sensor is used to determine the preoperative and intraoperative intra-abdominal pressures after reduction of the hernia to the cavity. These values should be <15 mm Hg in order to minimise the risk of compartment syndrome or abdominal hyperpressure (23).

This measurement is performed after abdominal closure and therefore serves as a validation tool for the volumetric and respiratory estimations used in the protocol.

However, it represents a limitation, as intra-abdominal pressure cannot be continuously monitored during the reduction process while the abdomen is open.

Consequently, transient increases in intra-abdominal pressure during reduction cannot be completely excluded, and surgical decision-making must also rely on preoperative planning, respiratory simulation and intraoperative clinical judgement.

In our initial experience, this protocol has been applied in 15 selected patients, with favourable short-term feasibility and without major early postoperative respiratory complications. However, because the sample size is small and follow-up is limited, these observations are presented only as preliminary feasibility data and not as proof of clinical efficacy or validation of the proposed thresholds.

## Discussion

This discussion has been expanded to emphasize that the proposed protocol is not intended to compete with established abdominal wall reconstruction strategies, but to add a respiratory-risk estimation layer to existing volumetric planning.

According to the European Hernia Society (24), a giant hernia is an abdominal wall defect with a diameter of more than 10 cm.

This type of hernia is challenging for surgeons due to the possibility of postoperative repercussions affecting the patient's abdominal and respiratory systems. Accordingly, treatment must be individualised and performed by an experienced medical team.

In such cases, the first requirement is to conduct a thorough assessment of the patient, including a detailed medical history review and a complete physical evaluation. Additionally, diagnostic tests such as ultrasound, CT scan and/or MRI should be performed to obtain a clear picture of the hernia and its impact on the abdominal organs. In this regard, CT scans are often used to calculate the volume of the hernia and the abdominal cavity (8), thus enabling the medical team to determine the relationship of volumes at which hernia reduction might cause problems, since the main drawback of this technique is its two-fold impact on the abdominal and respiratory systems.

Within the abdomen, the pressure generated by the reduced volume can give rise to compartment syndrome, which is defined by Kron (25) as the result of a progressive undetected increase in intra-abdominal pressure that provokes a series of alterations in different organs and which can lead to multi-organ failure.

At the respiratory level, the abdominal cavity is anatomically related to the rib cage through the diaphragm muscle. Using animal and human models, studies have shown that an average of 50% (range 25%–80%) of intra-abdominal pressure is transmitted to the thoracic cavity (26). Intra-abdominal hypertension generates a series of complex physiological changes in the cardiopulmonary system that can lead to postoperative complications such as acute respiratory failure and, in the worst case, respiratory distress syndrome (27).

In view of these potential complications, our protocol calls for an initial evaluation, followed by a CT scan to determine the relevant volumes. As indicated by Tanaka (8), the safety threshold in hernias is considered to be 25% of the volume of the abdominal cavity, since within this margin Tanaka's group observed no abdominal complications in any of the patients who underwent this surgery.

This approach is intended to avoid abdominal complications. In addition, we recommend a further precaution, that of considering the possible impact on the respiratory system of hernia reduction. A fundamental aspect of the protocol, therefore, is to consider the status of the patient's respiratory system following reduction of the hernia, by performing respiratory function tests with a progressive reduction of the abdominal cavity.

In our view, this combined approach, addressing both abdominal and respiratory concerns, is essential to optimise the management of a giant hernia. Given the necessary information before the operation takes place, appropriate steps can be taken. For example, if VHC/VAC >25% (risk of abdominal complications) or FEV1AR-TLCAR <60% (risk of respiratory complications), the physician can act preoperatively to increase the volume of the abdominal cavity and thus modify these parameters to improve the prognosis.

It is essential, therefore, in these cases to consider appropriate preoperative techniques to modify the cavity volume. For this purpose, the current literature indicates general agreement that infiltration with botulinum (16) toxin and the creation of pneumoperitoneum (17) are the most effective preoperative techniques.

In this regard, Hurtado (16) conducted a study of twelve patients who received botulinum toxin treatment. After four weeks of clinical and tomographic follow-up, the average reduction of the hernial defect was 5.25 ± 2.32 cm. Primary closure was achieved in half of the patients and in the remainder, closure was achieved with separation of components and no recurrences at nine months.

Botulinum toxin alone may be sufficient when the main limitation is lateral muscle retraction and the respiratory simulation remains favourable. Progressive pneumoperitoneum alone may be useful when the predominant problem is loss of domain and abdominal cavity adaptation. Combined botulinum toxin and progressive pneumoperitoneum is reserved for patients in whom both myofascial elongation and cavity expansion are required. The additional value of the present protocol is that these indications are not based solely on defect size or the Tanaka ratio, but also on predicted respiratory tolerance after reduction.

The use of pneumoperitoneum was first described in 1941 by Goñi Moreno (28) and later by other authors, in the United States (29). This technique consists of introducing air into the abdominal cavity, in several sessions, to generate progressive distension. The aim of this approach is to match the lung volume to the abdominal volume, and thus achieve closure without tension. There is no clear agreement in the literature on the amount of air that should be insufflated or the number of sessions to be given. In most cases, however, 500–1,000 cc of air is supplied, in 7–14 sessions. Our protocol calls for these values to be established in accordance with the estimated hernia volume to be reduced, as in our opinion it is important to individualise these cases. In 2018, Bueno Lledó (5) reported the cases of 100 patients who had been preoperatively prepared with progressive pneumoperitoneum and botulinum toxin. The average VHC/VAC ratio was significantly reduced (by 16.6%) and closure was completed in 97% of cases, with eight recurrences at 62 months.

Other researchers, too, have described the advantages of using pneumoperitoneum in the treatment of this type of hernia (30–32) and support the use of CT to evaluate the resulting increase in the length of the abdominal wall muscle (30). In our protocol, the CT scan enables the surgeon to assess the effectiveness of the preoperative Botox and/or pneumoperitoneum and thus to determine whether the relevant parameters have been corrected (i.e., VHC/VAC <25% and FEV1AR-TLCAR >60%).

For the surgical intervention, various techniques have been proposed for separating the muscle and fascial layers of the abdominal wall to facilitate reconstruction on the midline. Nevertheless, there is widespread agreement regarding the components separation technique described in a 1990 paper by Ramírez (33). This technique was subsequently developed and modified, and the Rives-Stoppa sublay technique for mesh placement (18–20) is currently considered the gold standard for treating this type of hernia. With this method, tension is released from the abdominal wall and the mesh is inserted without coming into contact with the intra-abdominal contents, whilst respecting the functionality of the abdominal wall as much as possible.

Anterior component separation, posterior component separation and TAR should be selected according to defect morphology, previous surgery, contamination, skin and soft-tissue quality, and the possibility of achieving fascial closure without excessive tension. TAR is favoured in this protocol when posterior component release can increase abdominal capacity while allowing retromuscular mesh placement and avoiding direct visceral contact whenever possible.

A literature review by Cornette (34) concluded that the rate of hernia recurrence after a Rives-Stoppa intervention was 11.3% at 22 months of follow-up. For Rives-Stoppa + TAR, the corresponding rate was 5.3% at 17 months of follow-up.

The protocol we present systematises the indications for the surgical technique to be used according to the preoperative values obtained for VHC/VAC and FEV1AR-TLCAR. The standard techniques for minor hernias are only used when VHC/VAC <25% + FEV1AR-TLCAR >60%. By contrast, if after preoperative preparation, components separation and TAR, the VHC/VAC value remains above 25% and/or the FEV1AR-TLCAR values remain <40% or 40%–60% with E-, the volume transposition technique must be applied, as these patients have very limited tolerance to other techniques (by using mesh to expand the abdominal cavity, we can calculate the volume increase that can be achieved without impairing the respiratory function). All other cases, in principle, can be addressed by means of the Rives-Stoppa technique.

In selected patients, staged reconstruction or watchful waiting may be safer than immediate definitive reconstruction, particularly when frailty, cardiopulmonary disease, uncontrolled obesity, active smoking, malnutrition or poor functional capacity make the predicted benefit uncertain. This point is essential because the protocol is a decision-support tool and does not replace clinical judgement.

Finally, it should be noted that intra-abdominal pressure can be measured indirectly by using a Foley catheter to record the pressure exerted on the urinary bladder. This can also be done with a nasogastric tube inserted into the oesophagus (measuring the vena caval pressure) or with an intraperitoneal catheter. However, we believe it most useful to obtain this measurement in the bladder as it provides a control mechanism to validate the measurements obtained previously.

## Limitations

The main limitation of this manuscript is the absence of definitive prospective validation. The proposed thresholds for VHC/VAC and FEV1AR/TLCAR are based on published volumetric concepts, adapted respiratory risk frameworks and institutional experience, but they require validation in a larger cohort of patients undergoing abdominal wall reconstruction.

The respiratory simulation test also requires further assessment of reproducibility, interobserver variability, patient tolerance and correlation with postoperative pulmonary function. The simplified volumetric formulas may be inaccurate in irregular hernias compared with segmentation-based CT volumetry.

Intravesical pressure measurement after closure is useful as a safety check, but it cannot continuously detect transient pressure changes during visceral reduction while the abdomen is open. These limitations must be considered when applying the protocol.

## Conclusions

The protocol we describe enables a very approximate calculation to be made of the volumes to be reintroduced into the abdominal cavity and the consequences of this action on the respiratory and abdominal functions, thus enabling control of the postoperative consequences in both directions, and minimising the risk involved in managing these volumes.

This study makes an important contribution to understanding the respiratory and abdominal implications for patients who require surgery to treat a giant hernia. By applying this protocol, valuable information can be obtained to guide clinical decision making, thus improving outcomes for this patient population.

To date, our early experience suggests feasibility in selected patients, but further prospective study with a larger number of cases is required to confirm the statistical validity, safety and predictive performance of the protocol. These results will be presented in a forthcoming validation study.

## Data Availability

The original contributions presented in the study are included in the article/Supplementary Material, further inquiries can be directed to the corresponding author.
